# Phoenix Sepsis Score and Risk of Attributable Mortality in Children With Cancer

**DOI:** 10.1001/jamanetworkopen.2024.15917

**Published:** 2024-06-10

**Authors:** Joshua Wolf, Zachary Rubnitz, Asya Agulnik, Jose Ferrolino, Yilun Sun, Li Tang

**Affiliations:** 1 Department of Infectious Diseases, St Jude Children’s Research Hospital, Memphis, Tennessee; 2 Department of Internal Medicine, University of Utah School of Medicine, Salt Lake City; 3 Department of Global Pediatric Medicine, St Jude Children’s Research Hospital, Memphis, Tennessee; 4 Department of Biostatistics, St Jude Children’s Research Hospital, Memphis, Tennessee

## Abstract

This prognostic study analyzes the accuracy of the Phoenix Sepsis Score for the classification of attributable mortality risk in children with cancer presenting to the intensive care.

## Introduction

Children receiving treatment for cancer are at high risk of sepsis-related death.^1,2^ In patients with sepsis, identifying those at greatest mortality risk is essential to target interventions, research, and counseling.^3^ However, general pediatric sepsis scoring systems perform poorly in children with cancer in high-income countries.^1,3^ This might be due to differences in clinical presentation or pathways to attributable death.

The newly developed Phoenix Sepsis Score, developed from 218 839 suspected-infection episodes, is recommended for all pediatric populations.^3^ However, children with cancer may have preexisting organ dysfunction affecting score performance, pediatric oncology subgroup analyses are unpublished, and the score has not been validated in pediatric oncology.^3,4^

## Methods

This prognostic study was approved by the St Jude Children’s Research Hospital institutional review board with waiver of consent because it was regarded as minimal risk. This study followed the Standards for Reporting of Diagnostic Accuracy (STARD) reporting guideline.

We used an existing retrospective cohort to evaluate the Phoenix Sepsis Score in children receiving treatment for cancer admitted to the intensive care unit (ICU) with suspected infection; the performance of Pediatric Risk of Mortality 3 (PRISM3), Pediatric Sequential Organ Failure Assessment (pSOFA), quick SOFA (qSOFA), and Paediatric Logistic Organ Dysfunction 2 (PELOD2) scores have been previously described.^1^ Briefly, we collected data for patients receiving cancer therapy (excluding hematopoietic cell therapy [HCT]), admitted to the ICU at St Jude Children’s Research Hospital between 2013 to 2019 with suspected infection. We calculated the Phoenix Sepsis Score using worst values recorded within 24 hours after the ICU admission, and then recalculated excluding platelet count (PHO-Phoenix).^4^ We estimated area under the receiver operating characteristic curve (AUROC) for classification of outcomes, including attributable mortality, definitely attributable mortality, all-cause mortality, and prolonged ICU stay (ie, more than 7 days).^1,5^ AUROCs were numerically compared with PRISM3, pSOFA, qSOFA, and PELOD2 scores.^1^ Additional methods are included in the eMethods in Supplement 1. Data were analyzed from January to February 2024. Calculations were made using R version 4.3.1 (R Foundation for Statistical Computing). Tests were 2-sided, and statistical significance was set at *P* < .05.

## Results

Of 207 episodes, 171 episodes in 143 participants met inclusion criteria (Table). The median (IQR) age was 10.3 (3.8-14.8) years; 60 (42.0%) participants were female; 81 (56.6%) had leukemia, and 62 (43.4%) had solid tumors or brain tumors. Race was self-reported, and 4 patients (2.8%) were Asian, 19 patients (13.3%) were Black, 1 patient (0.7%) was American Indian or Alaska Native, 107 patients (74.8%) were White, and 12 patients (8.4%) were more than 1 race. There were 17 cases of all-cause mortality (9.9%), 13 cases of attributable mortality (7.6%), 9 cases of definitely attributable mortality (5.3%), and 37 episodes (21.6%) classified as a prolonged ICU stay.

**Table.** Participant Characteristics (N = 143)

Characteristic	Participants, No. (%)
Sex	
Female	60 (42.0)
Male	83 (58.0)
Age, median (IQR), y^a^	10.3 (3.8-14.8)
Self-reported race	
American Indian or Alaska Native	1 (0.7)
Asian	4 (2.8)
Black	19 (13.3)
White	107 (74.8)
≥1 Specified race	12 (8.4)
Malignancy type	
Hematologic	81 (56.6)
Solid or brain tumor	62 (43.4)
HCT ever	4 (2.8)
BMI, mean (SD)^b^	20.3 (5.8)

Abbreviations: BMI, body mass index (calculated as weight in kilograms divided by height in meters squared); HCT, hematopoietic cell therapy.

^a^ Age in years at first included episode.

^b^ BMI at first included episode.

The 24-hour Phoenix Sepsis Score was significantly associated with all outcomes, and AUROC was numerically higher than other scores for attributable and definitely attributable mortality (Figure). Excluding thrombocytopenia did not improve discrimination.

**Figure.**
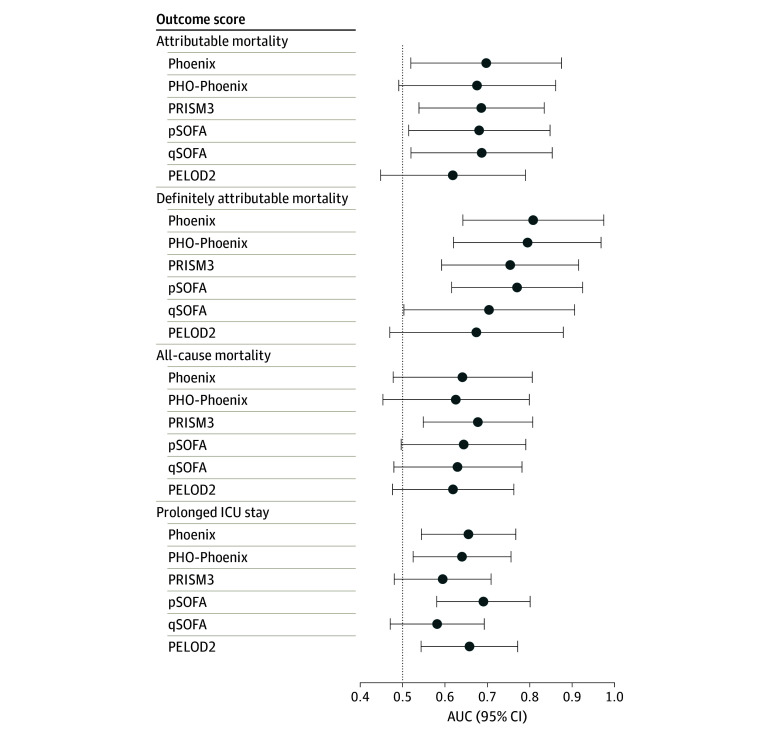
Performance of Phoenix Sepsis Score and Comparators for Prediction of Adverse Outcomes in Children With Cancer Admitted to the Intensive Care Unit With Suspected Infection PHO-Phoenix was the Phoenix Sepsis Score calculated excluding platelet count to account for possible confounding by chemotherapy-related thrombocytopenia. AUC indicates area under the curve; PELOD2, Paediatric Logistic Organ Dysfunction 2; PRISM3, Pediatric Risk of Mortality 3; pSOFA, Pediatric Sequential Organ Failure Assessment; qSOFA, quick SOFA.

At a cutoff of 2 points or more, the Phoenix Sepsis Score cutoff classified 129 episodes (75%) as sepsis; the sensitivity and specificity were 77% (95% CI, 51%-94%) and 25% (95% CI, 28%-52%) for attributable mortality and 89% (95% CI, 60%-99%) and 25% (95% CI, 19%-32%) for definitely attributable mortality, respectively. A cutoff of 4 points or more, selected by inspection of data, had a sensitivity and specificity of 69% (95% CI, 42%-89%) and 72% (95% CI, 64%-78%) for attributable mortality, and 89% (95% CI, 60%-99%) and 72% (95% CI, 64%-78%) for definitely attributable mortality. Positive predictive value for attributable mortality was 8% (95% CI, 4%-14%) at the cutoff of 2 points or more and 17% (95% CI, 8%-29%) at the cutoff of 4 points or more.

## Discussion

In this prognostic study, the Phoenix Sepsis Score was associated with mortality and prolonged ICU stay and numerically outperformed existing scores for attributable mortality. Although specificity was low, it improved at a higher post hoc cutoff. This study has limitations, including the small sample size, retrospective data collection, missing data, and exclusion of patients with HCT. Despite this, discrimination was very good for definitely attributable mortality and moderate for attributable mortality.^6^

Relatively poor performance of sepsis scores for attributable mortality in this population may be caused by delayed death and/or preexisting subclinical multiorgan dysfunction from cancer or chemotherapy.^5^ However, the temptation to focus only on definitely attributable mortality should be resisted as it might underestimate true sepsis-related mortality.

These findings suggest that the Phoenix Sepsis Score accurately classified the risk of definitely attributable mortality in children receiving treatment for cancer who were admitted to the ICU with suspected infection. More research is needed to validate these findings, evaluate population-specific cutoffs, and identify approaches that predict delayed attributable mortality.

## Notes

Supplement 1.
eMethods.

Supplement 2.
Data Sharing Statement
