# Supplementary material for: Phoenix Sepsis Score and Risk of Attributable Mortality in Children With Cancer
Source: JAMA Netw Open. 2024 Jun 10;7(6):e2415917. doi: 10.1001/jamanetworkopen.2024.15917 (PMC11165374; doi:10.1001/jamanetworkopen.2024.15917)
Supplement: Supplement 1. — eMethods. [file jamanetwopen-e2415917-s001.pdf]

Supplemental Online Content

Wolf J, Rubnitz Z, Agulnik A, Amadeo Ferrolino J, Sun Y, Tang L. Phoenix Sepsis Score and classifying risk of attributable mortality in children with cancer. *JAMA Netw Open*. 2024;7(6):e2415917. doi:10.1001/jamanetworkopen.2024.15917

eMethods.

This supplemental material has been provided by the authors to give readers additional information about their work.

© 2024 Wolf J et al. *JAMA Network Open*.

eMethods

Participants were all receiving treatment for cancer at St. Jude Children's Research Hospital, a pediatric comprehensive cancer center in Memphis, TN, USA. All 207 episodes of ICU admission with suspected infection included in the earlier convenience cohort were evaluated for inclusion in this substudy, and episodes were excluded if the participant was >18 years of age at the time of ICU admission, since development of the Phoenix Sepsis Score was restricted to this age group. Patients who had received hematopoietic cell therapy [HCT] were excluded if this was their most recent treatment for cancer, but were included if they had subsequently undergone further cancer-directed therapy, such as chemotherapy for relapsed leukemia.

Participant characteristics were reported for the first included episode. No patients were lost to follow-up. Self-reported Race was included because some infections are reported to be more common in specific racial groups.[1] Suspected infection was defined as collection of a blood culture plus initiation or modification of antibiotic therapy. The study was approved by the St. Jude Institutional Review Board prior to data abstraction.

All scores were calculated using the worst value recorded by 24 hours after ICU admission; values; values recorded for up to 6 hours prior to ICU admission were also included. All data were available at the time of score calculation. Participants were admitted to the ICU from the inpatient ward or clinics, as St. Jude does not have an emergency room. Patients transferred to ICU at St. Jude with onset of sepsis at another institution were excluded from the cohort. As a sensitivity analysis, we re-calculated the Phoenix Sepsis Score excluding platelet count (termed PHO-Phoenix for the purposes of this study) to account for possible confounding by chemotherapy-induced thrombocytopenia.[2] New onset of organ dysfunction within the first 24 hours of ICU admission was presumed to be sepsis-related.

The outcomes included attributable mortality (comprising death ≤60 days or before ICU discharge, without *resolution* of new onset organ dysfunction defined by consensus criteria), definitely-attributable mortality (attributable mortality without *improvement* in new onset organ dysfunction defined by consensus criteria), all-cause mortality (death ≤60 days or before ICU discharge), and prolonged ICU stay (>7 days). Participants with mortality prior to 7 days were classified as prolonged ICU stay to prevent survivorship bias.[3,4] AUROCs were numerically compared to the PRISM3, pSOFA, qSOFA, and PELOD2 scores calculated using worst values at 24 hours; these had been previously published and were recalculated for this age-restricted subcohort.[4] Data not available in the electronic health record were presumed to be normal.

© 2024 Wolf J et al. JAMA Network Open.

References

1. Inaba H, Pei D, Wolf J, et al. Infection-related complications during treatment for childhood acute lymphoblastic leukemia. *Ann Oncol.* 2017;28(2):386-392.
2. Sanchez-Pinto LN, Bennett TD, DeWitt PE, et al. Development and Validation of the Phoenix Criteria for Pediatric Sepsis and Septic Shock. *Jama.* 2024.
3. Weiss SL, Balamuth F, Hensley J, et al. The Epidemiology of Hospital Death Following Pediatric Severe Sepsis: When, Why, and How Children With Sepsis Die. *Pediatr Crit Care Med.* 2017;18(9):823-830.
4. Rubnitz Z, Sun Y, Agulnik A, et al. Prediction of attributable mortality in pediatric patients with cancer admitted to the intensive care unit for suspected infection: A comprehensive evaluation of risk scores. *Cancer medicine.* 2023;12(23):21287-21292.

© 2024 Wolf J et al. JAMA Network Open.
