# Supplementary material for: Phoenix Sepsis Score and Risk of Attributable Mortality in Children With Cancer
Source: JAMA Netw Open. 2024 Jun 10;7(6):e2415917. doi: 10.1001/jamanetworkopen.2024.15917 (PMC11165374; doi:10.1001/jamanetworkopen.2024.15917)
Supplement: Supplement 2. — Data Sharing Statement [file jamanetwopen-e2415917-s002.pdf]

Data Sharing Statement

Wolf. Phoenix Sepsis Score and Risk of Attributable Mortality in Children With Cancer. *JAMA Netw Open*. Published June 10, 2024. doi:10.1001/jamanetworkopen.2024.15917

Data
Data available: No
